# Peptide Fingerprinting of Alzheimer's Disease in Cerebrospinal Fluid: Identification and Prospective Evaluation of New Synaptic Biomarkers

**DOI:** 10.1371/journal.pone.0026540

**Published:** 2011-10-26

**Authors:** Holger Jahn, Stefan Wittke, Petra Zürbig, Thomas J. Raedler, Sönke Arlt, Markus Kellmann, William Mullen, Martin Eichenlaub, Harald Mischak, Klaus Wiedemann

**Affiliations:** 1 Department of Psychiatry, University Hospital Hamburg-Eppendorf, Hamburg, Germany; 2 Mosaiques Diagnostics GmbH, Hannover, Germany; 3 University of Applied Sciences, Bremerhaven, Germany; 4 Department of Psychiatry, Foothills Hospital, University of Calgary, Calgary, Canada; 5 Thermo Fisher Scientific GmbH, Bremen, Germany; 6 BHF Glasgow Cardiovascular Research Centre, University of Glasgow, Glasgow, United Kingdom; Thomas Jefferson University, United States of America

## Abstract

**Background:**

Today, dementias are diagnosed late in the course of disease. Future treatments have to start earlier in the disease process to avoid disability requiring new diagnostic tools. The objective of this study is to develop a new method for the differential diagnosis and identification of new biomarkers of Alzheimer's disease (AD) using capillary-electrophoresis coupled to mass-spectrometry (CE-MS) and to assess the potential of early diagnosis of AD.

**Methods and Findings:**

Cerebrospinal fluid (CSF) of 159 out-patients of a memory-clinic at a University Hospital suffering from neurodegenerative disorders and 17 cognitively-healthy controls was used to create differential peptide pattern for dementias and prospective blinded-comparison of sensitivity and specificity for AD diagnosis against the Criterion standard in a naturalistic prospective sample of patients. Sensitivity and specificity of the new method compared to standard diagnostic procedures and identification of new putative biomarkers for AD was the main outcome measure. CE-MS was used to reliably detect 1104 low-molecular-weight peptides in CSF. Training-sets of patients with clinically secured sporadic Alzheimer's disease, frontotemporal dementia, and cognitively healthy controls allowed establishing discriminative biomarker pattern for diagnosis of AD. This pattern was already detectable in patients with mild cognitive impairment (MCI). The AD-pattern was tested in a prospective sample of patients (n = 100) and AD was diagnosed with a sensitivity of 87% and a specificity of 83%. Using CSF measurements of beta-amyloid1-42, total-tau, and phospho_181_-tau, AD-diagnosis had a sensitivity of 88% and a specificity of 67% in the same sample. Sequence analysis of the discriminating biomarkers identified fragments of synaptic proteins like proSAAS, apolipoprotein J, neurosecretory protein VGF, phospholemman, and chromogranin A.

**Conclusions:**

The method may allow early differential diagnosis of various dementias using specific peptide fingerprints and identification of incipient AD in patients suffering from MCI. Identified biomarkers facilitate face validity for the use in AD diagnosis.

## Introduction

In an aging population dementias are a serious threat. Currently 30 million people worldwide suffer from Alzheimer's disease (AD) and the World Health organization projects that this number will triple over the next 20 years [1]. The cumulative incidence of AD has been estimated to rise from about 5% by age 70 to 50% by age 90 [2]. The clinical diagnosis of dementias is established late in the course of the disease process with poor sensitivity and specificity [3]–[5]. According to current diagnostic criteria, AD cannot be diagnosed before the disease has progressed so far that clinical dementia is present. The disease process, however, probably starts 20–30 years before first clinical signs emerge [6]. Hence we are in need of new diagnostic tools that are capable of detecting pre-clinical signs of neurodegenerative disorders. Recently different new analytical proteomic technologies like mass spectrometry coupled with protein separation or protein microarrays that can be applied on cerebrospinal fluid (CSF) have been developed to study proteins in neuroscience [7]. Due to the intimate relation between brain function and CSF composition, pathological brain-processes are more likely to be reflected in CSF than in other body-fluids (e.g. blood or urine). Since more than 70% of the CSF-proteins are isoforms of albumin, transferrin and immunoglobulines [8], and due to technical limitations only few studies have focused on the composition of proteins in CSF in the past [9]. Nonetheless an enormous wealth of information regarding pathological processes should be present in the less abundant CSF-proteins and the identification of changes in CSF composition at that level beside the current disease models would promote the understanding of the various dementias and their fundamental pathological processes. Such valid new biomarkers for AD could also serve as surrogate markers in detecting treatment effects while any earlier identification of AD patients is another goal to enable the development of treatments that stop or postpone the disease processes.

We report proteome analysis of CSF using capillary electrophoresis coupled to an electrospray ionisation time of flight mass spectrometer (CE-MS) and its potential use in the diagnosis of AD and other dementias. This approach allows the comprehensive analysis of low molecular weight peptides and protein fragments present in biological fluids in a single time-limited step. The method was already successfully applied to the examination of human urine for the differential diagnosis of renal diseases [10]–[13], the diagnosis of prostate or urothelial cancer [14]–[16], ureteropelvic junction obstruction [17], and rejection of renal transplants [18] demonstrating the broad application spectrum of this new technique that also allows the comparison of biomarker sequencing data by use of different mass spectrometer types [19]. In addition we already implemented the recommendations for studies in clinical proteomics that were recently formulated by experts in the field [20] to avoid pitfalls and circumvent methodological problems that became apparent in earlier studies in this fast developing field of science.

## Methods

### Ethics Statement

The study was approved by the ethics committee of the “Ärztekammer Hamburg, Germany” and all patients and/or their relatives gave written informed consent and all clinical investigations have been conducted according to the principles expressed in the Declaration of Helsinki. Furthermore, the University Hospital Hamburg-Eppendorf has carried out all investigations according to international Good Laboratory Practice (GLP) and Good Clinical Practice (GCP) standard.

### Patient characteristics

Between April 2002 and December 2005 176 patients referred to the memory clinic of the University Hospital Hamburg-Eppendorf were recruited for this study. All patients underwent a diagnostic work-up and were diagnosed according to ICD-10 and the National Institute of Neurological and Communicative Disorders and the Stroke-Alzheimer's Disease and Related Disorders Association criteria (NINCDS-ADRDA) to identify patients with vascular dementia [21], [22]. MCI diagnoses were made according to the criteria of Petersen [23] and FTD was diagnosed according to the Lund–Manchester criteria [24]. The structure of cognitive dysfunction was assessed with a neuropsychological test battery allowing the identification of MCI subtypes. Distinction of MCI and dementia was based on CDR rating (see **Table S3** for neuropsychological tests). Patients underwent lumbar puncture for diagnostic purposes. In a first step CSF of 17 control samples of cognitively healthy volunteers (these persons underwent a lumbar puncture in connection with a knee arthroscopy and agreed to obtain an additional cognitive testing apart from allowing to collect some CSF during peridural anaesthesia), 34 samples of patients with a clinical diagnosis of sporadic AD, 12 samples of patients with FTD and 13 samples of patients with schizophrenia (**Table 1**) was analyzed to establish disease specific peptide patterns. The clinical diagnoses of these training cases were supported by laboratory and image data from magnetic resonance imaging (MRI) and positron emission tomography (PET). The training set patients were selected to have no other major medical comorbidity. In a second step 100 patients (51 female, 49 male; mean age 65.3±12.3 years) (**Table S2**) with memory complaints were recruited for a prospective sample cohort, CSF was analysed to obtain a diagnosis and patients were clinically followed. These patients suffered from various dementias, MCI or memory complains of other causes. A few had further medical comorbidities like hypertension or diabetes type II or a history of insults or encephalitis.

**Table 1:** Demographic data of training sets.

Group	Number	Male	Female	Age [years±SD]	MMSE [mean±SD]
**Controls**	17	13	4	58.9±11,5	30.0±0.0
**AD Training set**	34	14	20	69.0±6.5	21.2±5.1
**FTD Training set**	12	4	8	65.6±7.9	22.4±2.5
**Schizophrenia set**	13	5	8	38.1±14.4	29.6±0.7

Baseline data of training sets for cognitively healthy controls and trainings sets for AD, FTD and schizophrenia. Given are numbers for gender, and the averages for age and the scores of the Mini Mental State Examination (MMSE).

### Cerebrospinal fluid samples

Cerebrospinal fluid (CSF) was obtained by lumbar puncture in a sitting position according to standard procedures [25]. After collection of the 4 mL CSF for routine diagnosis, additional 5 ml of the CSF was sampled for this study into a polypropylene test tube. The CSF was centrifuged immediately after collection (1600 g, 4°C, 15 min), aliquoted into polypropylene test tubes (each aliquot, 750 µL), frozen within 30–40 min after the puncture and stored at −80°C until use. The CSF was at no time thawed/refrozen. After thawing, 700 µL of CSF were diluted with 700 µL buffer (pH 10.5; 2 M urea, 100 mmol/L NaCl, 0.0125% NH_3_; Sigma-Aldrich, Taufkirchen, Germany) and ultracentrifugated using Centrisart ultrafiltration devices (cut off 20 kDa, Sartorius, Göttingen, Germany) at 4°C until 1.1 mL of filtrate was obtained. Subsequently the filtrate was applied onto a PD-10 desalting column (Amersham Bioscience, Uppsala, Sweden) equilibrated in 0.01% NH_4_OH in HPLC-grade in water (Roth, Germany) to decrease matrix effects by removing urea, electrolytes, salts, and to enrich peptides present. Finally the eluate was lyophilized stored at 4°C and resuspended in 10 µL HPLC-grade water before CE-MS analysis.

Since blood contaminations may affect the proteome of CSF [26] we controlled our CSF samples for traces of haemoglobin and the presence of erythrocytes by microscopy of a centrifuged CSF sample. Using these two methods we can exclude any blood contamination of our CSF samples above 0.001% by that controlling a potential serious confounder of CSF biomarker studies with regard to proteins abundant in plasma.

### Immunochemistry

The CSF levels of Aß42, total tau, and phospho_181_-tau were measured using commercial ELISAs (Innogenetics, Ghent, Belgium) according to the manufacturer's protocol. Cut-off values for AD suspicious biomarker concentrations were >540 pg/ml for total-tau, >61 pg/ml for phospho_181_-tau and beta-amyloid1–42 values <240+1.18×total-tau pg/ml [4].

### CE-MS

CE-MS analysis was performed as described using a P/ACE MDQ (Beckman Coulter, Fullerton, USA) system on-line coupled to a Micro-TOF MS (Bruker Daltonic, Bremen, Germany) [15]–[17].

### Reproducibility and comparability

Analytical characteristics such as accuracy, mass accuracy, traceability and repeatability of the CE-MS application were recently described [15], [27]. Thus, here we report about additional precision tests to underline the validity of the analytical data. Initially the sampling procedure was validated by fractionated extraction of six aliquots of CSF from one patient. **Figure S1** shows the protein contour plots of 4 different fractions. Classification of each fraction resulted in the same and correct classification as AD. We re-measured 10 samples twice and again all samples were classified correctly (**Table S4**). We were also able to re-puncture one patient with an AD pattern. The AD pattern was found again and stable after one year. Finally the reproducibility of the CE-MS approach is emphasized (**Figure S2**). Distinct lines (four to six) are visible in the peptide pattern due to the charge per peptide, which can be ascribed to the peptide structure (number of basic amino acids) and consequently allow the comparison of biomarker sequencing data even by use of different mass spectrometer types coupled with CE or liquid chromatography (LC) [19].

### Data processing and cluster analysis

Mass spectral ion peaks representing identical molecules at different charge states were deconvoluted into single masses using MosaiquesVisu [27]. In addition the migration time and ion signal intensity (amplitude) were normalized using internal peptide standards [27], [28]. The resulting peak list characterizes each peptide by its molecular mass (in Dalton), normalized CE-migration time (in minutes), and normalized signal intensity. All detected peptides were deposited, matched, and annotated in a Microsoft SQL database, allowing further analysis and comparison of multiple samples (patient groups) (**Table S5**). Peptides within different samples were considered identical if the mass deviation was less than 100 ppm and the migration time deviation was less than 1 min.

To define biomarker patterns we employed the following stringent quality control and selection criteria to avoid artefacts as well as to attain high levels in reproducibility and specificity: 1) the MS peaks list must contain 800–1400 peptides; 2) the normalized mean signal amplitude of each marker peptide must be >25 counts in one diagnostic group; 3) the frequency of occurrence (FOC) of each candidate biomarker must be >40% in one of the groups defined above; 4) the difference in the FOC between the two groups must be either >30% or, 5) if the difference in the FOC is less than 30%, the mean amplitude in one diagnostic group must be >1.4-fold higher compared to the other group and the FOC of the selected amplitude biomarker must be >70% in the diagnostic group with the higher mean amplitude.

Employing these criteria, a list of pre-defined peptides was obtained by the comparison of all available data sets (e.g. control vs. AD, FTD, schizophrenia patients, respectively) with MosaCluster software package [13]. The pre-defined set of peptides was further validated by randomly excluding 30% of available samples. This procedure was repeated 5 times to utilize markers (preliminary biomarker pattern) with consistently high discriminative value. Subsequently the preliminary biomarker patterns were refined by employing an unadjusted p-value limit of 0.01 and using one-out cross-validation to attain high sensitivity and specificity. This approach resulted in the definition of biomarker patterns consisting of 10–20 biomarkers.

### Tandem sequencing

Tandem (MS/MS) sequencing was performed using Orbitrap instruments (Thermo Finnigan, Bremen, Germany). Orbitrap experiments were performed on a Dionex Ultimate 3000 nanoflow system connected to an LTQ Orbitrap hybrid mass spectrometer (Thermo Electron, Bremen, Germany) equipped with a nanoelectrospray ion source. Binding and chromatographic separation of the peptides took place on a fused silica nanocolumn (10 cm; 75-µm i.d.; C18, 5 µm; NanoSeparations, Nieuwkoop, Netherlands). The MS was operated in data-dependent mode to automatically switch between MS and MS/MS acquisition. Full scan MS spectra (from *m*/*z* 300–2000) were acquired in the Orbitrap with resolution *R = *60,000 at *m*/*z* 400 (target value of 500,000 charges in the linear ion trap).

### Statistical Analysis

To verify the diagnostic value of the potential biomarkers pre-selected by the criteria mentioned above, raw p-values (so called unadjusted p-values) were calculated using the natural logarithm transformed intensities and the Gaussian approximation to the t-distribution, both implemented as macros in the commercial statistical package SAS (SAS Institute, Cary, NC; www.sas.com). Estimates of sensitivity and specificity were calculated based on tabulating the number of correctly classified samples. The receiver operating characteristic (ROC) curve was obtained by plotting all sensitivity values (true positive fraction) on the y axis against their equivalent (1-specificity) values (false positive fraction) for all available thresholds on the x-axis (MedCalc Software, Mariakerke, Belgium, www.medcalc.be). The area under the ROC curve (AUC) was evaluated as it provides a single measure of overall accuracy that is not dependent upon a particular threshold. After the best suited biomarkers are selected disease specific biomarker patterns (model) were established and the internal sensitivity and specificity for the training set were determined by leave one-out cross-validation analysis as described [15].

Due to the difference of the mean age between the training sets for the control cohort and patients suffering on Alzheimer's disease we verified if the AD-pattern is related to age or not. For this purpose the control group was divided into two subgroups, whereas the first group consisted of controls aged 61 or younger (n = 10; mean age 54±12) and the second group of patients aged 63 or older (n = 7, mean age 65±4).

Adapting the AD-biomarker pattern to these two subgroups resulted in a misclassification rate of 43% after cross-validation. Hence the AD-biomarker pattern used in this study is not capable to discriminate between the two subgroups and therefore an age related inconsistency in the pattern is unlikely.

## Results

CSF-samples were investigated using CE-MS with the aim to define a panel of disease-specific biomarkers that allow the identification and prospective diagnostic labelling of patients with various dementias, mild cognitive impairment (MCI) and their separation from healthy controls (for demographic data see **Table 1**).

### Development of disease specific peptide patterns

In each sample we were able to tentatively identify >800 different peptides based on migration time and mass, with molecular weights ranging from 0.8 to 20 kDa. The data from the individual CE-MS analyses were compiled and specific biomarker patterns for AD, FTD and schizophrenia were developed with emphasis on a panel of biomarkers that should enable to detect AD-cases based solely on peptides in CSF.

**Figure 1:**
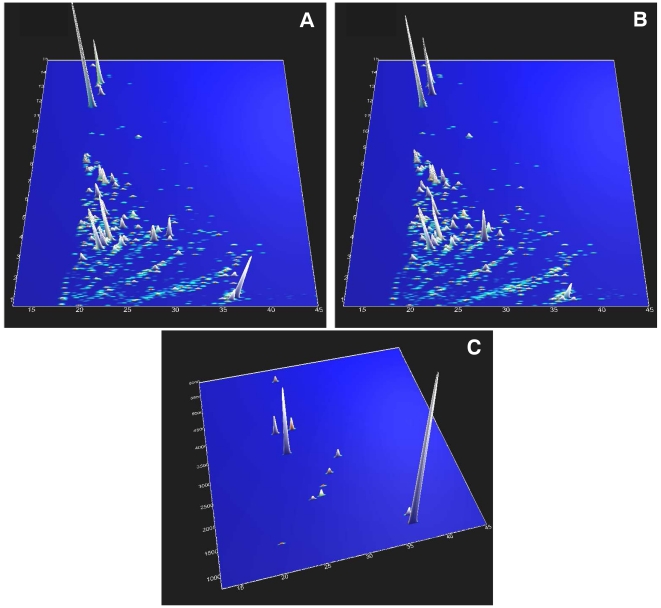
Contour-plots of the training set. **A**) Compiled 3-D protein contour plot from CSF samples of 34 patients with Alzheimer's disease. The normalized CE-migration time (in min) is plotted on the x-axis and the relative molecular mass (in kDa) on the y-axis. As a third dimension, the signal intensity is colour coded (blue lowest and white highest signal intensity). Each dot represents one peptide. **B**) Compiled 3-D protein contour plot for healthy controls (n = 17). **C**) Discriminative biomarker pattern for subjects suffering from Alzheimer's disease (n = 34). Depicted is a 3-D plot of 12 peptides that serve as specific biomarkers for AD brain damage. The normalized CE-migration time (in min) is plotted on the x-axis and the relative molecular mass (in Da) on the y-axis. As a third dimension, the signal intensity is colour coded.

**Figure 1A** shows the compiled data of 34 measurements of CSF from patients with sporadic Alzheimer's disease, while **Figure 1B** shows the data for the healthy controls from our training sets.

**Figure 2:**
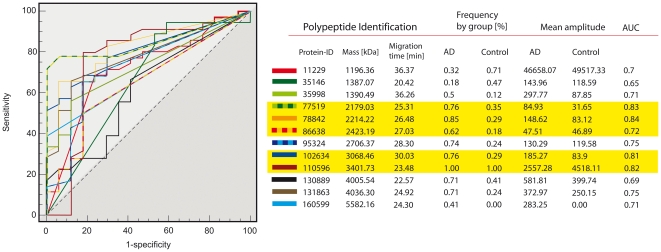
Peptide pattern identifying AD. Shown are the ROC analyses of all biomarkers. In the table unique internal protein-ID, mass, CE-migration time, observed frequency of occurrence, the corresponding mean amplitudes, and AUC values (as a biomarker quality measure) in the different groups for the final peptide panels. Five peptides, which are marked in yellow, are already identified (**Table 4**).

For the identification of AD specific peptides, analyses of these data with MosaCluster yielded 131 potentially biomarkers (**Table S1**), which fulfil the defined selection criteria (see Methods section “Data processing and cluster analysis”). Given the number of cases and controls available, the pattern of 131 potential biomarkers was refined by statistical testing and employing an unadjusted p-value limit of 0.01, resulting in a preliminary diagnostic pattern of 35 potential biomarkers (**Table S1**, see bold marked peptides). Subsequently the preliminary pattern was iteratively refined by eliminating one potential biomarker and utilizing leave-one-out cross-validation for verification (while maintaining high sensitivity and specificity). Leave-one-out cross validation uses a single sample as the validation data and the remaining samples as the training data. Each sample is used once as the validation data, giving an indication of how well the training set will perform when it is applied on unknown samples. This approach yielded a final diagnostic pattern (“AD-pattern”) based on 12 discriminatory peptides (**Figure 1C**). Utilizing support vector machines the AD-pattern allowed correct classification of 31/34 AD and 15/17 control samples, thus a sensitivity of 91% and specificity of 88%. The data including the AUC of the receiver operating characteristic (ROC) for these 12 biomarkers are shown in **Table 2** and **Figure 2**. The overall AUC for this biomarker pattern in the training set was calculated as 0.979 (95% CI: 0.893–0.997; p-value = 0.0001) pointing towards the potential of this multidimensional approach.

**Table 2:** Peptide pattern identifying AD.

Peptide identification			Frequency by group [%]		Mean amplitude		
Protein ID	Mass [Da]	CE-time [min]	AD	Control	AD	Control	AUC
11229	1196.36	36.37	0.32	0.71	46658	49517	0.70
35146	1387.07	20.42	0.18	0.47	144	119	0.65
35998	1390.49	36.26	0.50	0.12	298	88	0.71
**77519**	**2179.03**	**25.31**	**0.76**	**0.35**	**85**	**32**	**0.83**
**78842**	**2214.22**	**26.48**	**0.85**	**0.29**	**149**	**83**	**0.84**
**86638**	**2423.19**	**27.03**	**0.62**	**0.18**	**48**	**47**	**0.72**
95324	2706.37	28.30	0.74	0.24	130	120	0.75
**102634**	**3068.46**	**30.03**	**0.76**	**0.29**	**185**	**84**	**0.81**
**110596**	**3401.73**	**23.48**	**1.00**	**1.00**	**2557**	**4518**	**0.82**
130889	4005.54	22.57	0.71	0.41	582	400	0.69
131863	4036.30	24.92	0.71	0.24	373	250	0.75
160599	5582.16	24.30	0.41	0	283	0	0.71

Shown are unique internal protein-IDs, mass, CE-migration time, observed frequency of occurrence, the corresponding mean amplitudes, and AUC values (as a biomarker quality measure) for the final peptide panel. Five peptides, which are marked in bold, are already identified (see **Table 4**).

Subsequently this approach was also used to establish biomarker patterns for FTD (14 discriminatory peptides; data not shown) and schizophrenia (14 discriminatory peptides; data not shown). In the case that more than one disease specific pattern scores positive the use of differential diagnostic patterns is required for a final classification. Thus differential diagnostic patterns for AD versus FTD and AD versus schizophrenia based on the disease specific biomarkers have also been established.

### Blinded evaluation of prospectively collected samples

Using the defined patterns a blinded evaluation of additional prospective collected samples of 100 individuals followed. Whereas all non-AD samples in this study were used as disease controls, the blinded and prospective group consisted of samples from patients with multiple neurodegenerative disorders like MCI, vascular dementia, Parkinson's dementia, FTD, depression or other dementia syndromes. Given the need to differentiate against other forms of dementia and also other neuropsychological diseases, a hierarchical model for the classification of the blinded and prospectively collected samples was established. For this issue, in a first step the biomarker patterns for AD, FTD, and schizophrenia are applied onto the unknown sample for classification. If more than one disease-pattern scores positive differential diagnostic patterns are used for final classification. By this approach we circumvent the methodological problems of several investigations in proteomic research, which compare only two diagnostic groups [29], [30].

Our method using the defined peptide pattern showed a sensitivity of 87% and a specificity of 83% for the diagnosis of AD of the prospective samples (**Table S2**). Using CSF measurements of beta-amyloid1–42, total-tau and phospho_181_-tau, AD diagnosis had a sensitivity of 88% and a specificity of 67% in our clinical cohort. Interestingly, four patients diagnosed with primary progressive aphasia were classified as positive for both FTD and AD patterns.

Epidemiological studies have documented the accelerated rate of progression to dementia and AD in subjects with MCI. However, many MCI cases will never develop dementia [23]. To date there is no accepted clinical method to classify which MCI cases will progress to AD.

32 cases (17 male, 15 female; age: 66.1±6.6 years; MMSE: 27.8±1.9; values as mean ± SD) in the blinded set were originally diagnosed with MCI. After lumbar puncture the patients were clinically followed up for a mean of 57±7 months (mean ± SD; max 72, min 41 months). Twenty-one patients scored positive for the AD pattern, 11 patients scored negative for the AD pattern. The clinical outcome is shown in **Table 3**. In summary, the MCI cases with a positive AD pattern did clinically worse and showed a high conversion rate to AD. Furthermore, from 16 patients with a multi-domain or amnestic MCI, a diagnosis related to a higher risk of developing AD, 14 patients displayed a positive AD pattern in CSF. From the remaining 16 other MCI cases, only 7 patients showed a positive AD pattern. Using the Chi square test this difference is significant (p = 0.0255; p-value with continuity correction) pointing to an association of the former MCI subtypes with an AD pattern in CSF in our cohort.

**Table 3:** Clinical outcome of MCI cases by peptide pattern.

	Number	AD	OD	MCI	Remitter
Positve AD-Score	21	14	0	7	0
Negative AD-score	11	2	1	5	3

Clinical outcome of MCI cases (n = 32) followed up for a mean of 57 months. MCI cases with a positive score for the AD-pattern (n = 21) or a negative score for the AD-pattern (n = 11) according to our peptide panel. From 21 MCI patients originally identified as having a positive score for the AD pattern in CSF, only 7 patients are still clinically diagnosed with MCI, while 14 progressed into clinical AD. The other MCI group with negative score for the AD pattern showed a different outcome. Here only 2 patients progressed into AD so far, while 5 patients are still diagnosed with a stable MCI, one patient progressed into a vascular dementia (other dementia, OD), and 3 patients developed a complete remission of cognitive symptoms.

### Identification of biomarkers

In **Table 4** the identified peptide sequences out of the initial list of 131 potential AD biomarkers are shown: So far we identified 57 sequences deriving from 24 different protein precursors. We identified 2 fragments of amyloid beta A4 protein, 1 fragment of apolipoprotein E, 1 fragment of apolipoprotein J or clusterin, 6 fragments of amyloid-like protein 1 (APLP1), 1 fragment of amyloid-like protein 2 (APLP2), 1 fragment of collagen alpha 1(l) chain, 2 fragments of fibrinogen alpha chain, 1 fragment of complement C4-A, 1 fragment of son of sevenless homolog 2, 1 fragment of cystatin C, 1 fragment of cholecystokinin, 7 fragments of neurosecretory protein VGF, 7 fragments of proSAAS, 5 fragments of neuroendocrin protein 7B2, 7 fragments of chromogranin A, 3 fragments of secretogranin I, and 1 fragment each of segretogranin II–III. We found 1 fragment of integral membrane protein 2B, 1 fragment of osteopontin, 2 fragments of testican-1, and 2 fragments of brevican core protein. Finally, we also identified 1 fragment of phospholemman (FXYD1) and FXYD6.

**Table 4:** Sequence analysis.

Pr ID	Mass [Da]	CE-time [min]	Sequence	Protein name	start	stop	calc. mass [Da]
2065	1013.43	25.18	FMSDTREE	Secretogranin-1	430	437	1013.4124
3251	1083.55	25.61	FENKFAVET	Integral membrane protein 2B	254	262	1083.5237
7029	1158.62	27.02	EDVGSNKGAIIG	Amyloid beta A4 protein	693	704	1158.5881
9404	1183.66	27.49	DPPAIPPRQPP	Son of sevenless homolog 2	1171	1181	1183.6350
9730	1186.56	27.21	HELDSASSEVN	Osteopontin	273	287	1186.5102
17258	1250.61	27.59	DHDVGSELPPEG	ProSAAS	223	234	1250.5415
19245	1265.63	27.22	SpGPDGKTGPpGPA	Collagen alpha-1(I) chain	546	559	1265.5888
24081	1300.67	29.70	DELAPAGTGVSRE	Amyloid-like protein 1	568	580	1300.6259
24573	1306.74	22.16	VSPAAGSSPGKPPR	Cystatin C	21	34	1306.6993
30960	1349.67	28.25	SGEGDFLAEGGGVR	Fibrinogen alpha chain	22	35	1349.6212
37415	1404.73	29.49	HDVGSELPPEGVLG	ProSAAS	224	237	1404.6885
41974	1448.69	29.41	EAVEEPSSKDVME	Chromogranin-A	119	131	1448.6341
44123	1475.77	30.05	HDVGSELPPEGVLGA	ProSAAS	224	238	1475.7256
45572	1499.74	29.89	LDDLQPWHSFGAD	Amyloid beta A4 protein	615	627	1499.6681
45788	1500.71	22.99	SVPHFSDEDKDPE	Neuroendocrine protein 7B2	200	212	1500.6369
47143	1519.76	31.87	DHDVGSELPPEGVLG	ProSAAS	223	237	1519.7155
48140	1535.74	30.00	ADSGEGDFLAEGGGVR	Fibrinogen alpha chain	20	35	1535.6852
49413	1552.61	30.78	VTEDDEDEDDDKE	Testican-1	420	432	1552.5537
51940	1596.83	30.32	KVEQAVETEPEPEL	Apolipoprotein E	19	32	1596.7883
53060	1614.84	31.54	DELAPAGTGVSREAVSG	Amyloid-like protein 1	568	584	1614.7849
58375	1727.92	32.13	DELAPAGTGVSREAVSGL	Amyloid-like protein 1	568	585	1727.8690
60472	1771.97	30.02	SVNPYLQGQRLDNVVA	Neuroendocrine protein 7B2	182	197	1771.9217
62126	1816.98	33.53	DHDVGSELPPEGVLGALL	ProSAAS	219	240	1816.9207
62500	1820.06	32.23	DVGSELPPEGVLGALLRV	ProSAAS	221	242	1820.0044
64448	1867.72	33.07	AVTEDDEDEDDDKEDE	Testican-1	419	434	1867.6603
64930	1873.00	21.26	ALHPEEDPEGRQGRLLG	Brevican core protein	879	895	1872.9442
66400	1900.07	24.19	SVNPYLQGQRLDNVVAK	Neuroendocrine protein 7B2	182	198	1900.0167
66858	1907.99	21.60	ADPAGSGLQRAEEAPRRQ	Cholecystokinin	26	43	1907.9562
67973	1927.02	21.00	VAKKSVPHFSDEDKDPE	Neuroendocrine protein 7B2	196	212	1926.9323
71613	2028.17	21.62	SVNPYLQGQRLDNVVAKK	Neuroendocrine protein 7B2	182	199	2028.1116
71707	2029.13	20.15	ALHPEEDPEGRQGRLLGR	Brevican core protein	879	896	2029.0453
72233	2041.99	32.56	ELSAERPLNEQIAEAEED	Secretogranin-3	35	52	2041.9440
74137	2085.12	33.75	DELAPAGTGVSREAVSGLLIM	Amyloid-like protein 1	568	588	2085.0776
75685	2128.97	26.03	EGQEEEEDNRDSSMKLSF	Chromogranin-A	359	376	2128.8855
**77519**	**2179.03**	**25.31**	**SGELEQEEERLSKEWEDS**	**Chromogranin-A**	**322**	**339**	**2178.9553**
**78842**	**2214.22**	**26.48**	**AADHDVGSELPPEGVLGALLRV**	**ProSAAS**	**217**	**242**	**2214.1644**
79150	2222.16	26.60	STKLHLPADDVVSIIEEVEE	Neurosecretory protein VGF	459	478	2222.1318
84897	2377.25	28.07	DDPDAPLQPVTPLQLFEGRRN	Complement C4-A	1429	1449	2377.2026
**86638**	**2423.19**	**27.03**	**ESPKEHDPFTYDYQSLQIGGL**	**Phospholemman**	**21**	**41**	**2423.1281**
86817	2428.11	27.16	SSQGGSLPSEEKGHPQEESEESN	Secretogranin-1	293	315	2428.0262
88199	2471.30	35.56	DELAPAGTGVSREAVSGLLIMGAGGGS	Amyloid-like protein 1	568	593	2471.2326
88418	2475.21	27.09	HSGFEDELSEVLENQSSQAELK	Chromogranin-A	97	118	2475.1401
90848	2549.29	27.66	VGGLEEERESVGPLREDFSLSSSA	Amyloid-like protein 2	671	694	2549.2244
91583	2584.37	35.60	DELAPAGTGVSREAVSGLLIMGAGGGSL	Amyloid-like protein 1	568	595	2584.3167
94378	2684.32	21.38	SAAEKEKEMDPFHYDYQTLRIGG	FXYD6	19	41	2684.2541
**102634**	**3068.46**	**30.03**	**DQTVSDNELQEMSNQGSKYVNKEIQNA**	**Clusterin**	**22**	**49**	**3068.3993**
104636	3173.68	22.95	GRPEAQPPPLSSEHKEPVAGDAVPGPKDGSAP	Neurosecretory protein VGF	26	57	3173.5742
108011	3302.68	23.31	GRPEAQPPPLSSEHKEPVAGDAVPGPKDGSAPE	Neurosecretory protein VGF	26	58	3302.6044
**110596**	**3401.73**	**23.48**	**GRPEAQPPPLSSEHKEPVAGDAVPGPKDGSAPEV**	**Neurosecretory protein VGF**	**26**	**59**	**3401.6852**
115988	3595.77	23.89	PPGRPEAQPPPLSSEHKEPVAGDAVPGPKDGSAPEV	Neurosecretory protein VGF	24	59	3595.7907
116478	3614.82	22.01	GRPEAQPPPLSSEHKEPVAGDAVPGPKDGSAPEVRG	Neurosecretory protein VGF	26	61	3614.8077
119503	3685.86	22.21	GRPEAQPPPLSSEHKEPVAGDAVPGPKDGSAPEVRGA	Neurosecretory protein VGF	26	62	3685.8449
121774	3768.77	31.84	SGFEDELSEVLENQSSQAELKEAVEEPSSKDVME	Chromogranin-A	98	131	3768.7048
122653	3792.76	27.25	HSGFEDELSEVLENQSSQAELKEAVEEPSSKDVm	Chromogranin-A	97	130	3792.7160
126695	3921.82	27.74	HSGFEDELSEVLENQSSQAELKEAVEEPSSKDVmE	Chromogranin-A	97	131	3921.7586
139272	4319.85	23.64	DPADASEAHESSSRGEAGAPGEEDIQGPTKADTEKWAEGGGHS	Secretogranin-1	88	130	4319.8711
140319	4349.06	28.17	VPGQGSSEDDLQEEEQIEQAIKEHLNQGSSQETDKLAPVS	Secretogranin-2	527	566	4349.0419

Identified sequences of 57 out of the initial set of 131 putative AD biomarkers are listed. Shown are unique internal protein ID (Pr ID), molecular mass (in Dalton), CE-migration time (in minutes), sequence, originating protein name, and calculated mass (in Dalton). Peptides that are utilized in the final AD pattern are highlighted in bold letters.

We have satisfactory mass spectra of all 12 AD biomarkers, which were used in the AD pattern (see **Figure S3**). However, we are not able to identify all peptides. The greater challenge of peptide identification in comparison to protein identification of classical proteomic studies is the missing availability of peptide mass fingerprint information. Sequencing is hindered by several obstacles associated with MS sequencing of naturally occurring peptides (tryptic digests cannot be utilized because of a loss of connectivity to the original identification parameters [31]). Major obstacles are suboptimal use of proteomics search machines (like MASCOT or OMSSA) for naturally occurring peptides [32], [33] as well as the chemical nature (e.g. post-translational modifications) of the peptides that prevents successful sequencing [19]. To date, sequences could be obtained from 5 of the final 12 best-of-selection of AD biomarkers with the ID 78842: AADHDVGSELPPEGVLGALLRV, ProSAAS precursor [217–242]; ID 77519: SGELEQEEERLSKEWEDS, chromogranin A [322–339], ID 86638: ESPKEHDPFTYDYQSLQIGGL, phospholemman (FXYD1) [21]–[41]; ID 102634: DQTVSDNELQEMSNQGSKYVNKEIQNA, clusterin/apolipoprotein J [22]–[49]; and ID 110596: GRPEAQPPPLSSEHKEPVAGDAVPGPKDGSAPEV, neurosecretory protein VGF [26]–[59] (**Table 2**). Annotated mass spectra of these peptides are shown in **Figure S3**. Uniformly AD patients showed higher detection frequencies and amplitudes for most of these synaptic marker peptides in CSF that point toward alterations in vesicle maturation and transport in AD (**Table 4**). However, fragments of VGF, a peptide also found in synaptic vesicles, were detected with lower signal amplitude in AD patients.

A subset of the patient cohort (n = 61) could be screened for their apolipoprotein E (ApoE) allelic composition. About 50% carried an ApoE4 allele. The ApoE4 allele variant is a known risk factor for AD found in about 40–50% of all AD cases, while the ApoE4 allele frequency in a normal population is about 10% [34]. Although it is commonly not recommended as a screening test, a positive ApoE4 status predicts AD with a sensitivity of 80% when clinical symptoms of a dementia are present [34]. The allelic status of 27 from 34 patients with clinical AD from our training set could be determined to investigate if the discriminative value of the AD-specific biomarkers changes in relationship to the ApoE4 status. 18 of 27 genotyped AD-patients were showing a positive ApoE4 status with at least one copy. The discriminative value of the 12 biomarkers in the final panel for AD (**Figure 2**) does not change significantly in view to a positive ApoE4 status. In contrast the N-terminal apolipoprotein E fragment ([19]–[32]; KVEQAVETEPEPEL) originally included in the primary candidate list or the testican-1 fragments (**Table 4**) showed significantly better AUC values if patients carried the ApoE4 allele. We excluded affected biomarkers from the final list to avoid contamination with the already known risk factor due to the different allele frequencies in the diagnosis groups.

## Discussion

The urgent need of earlier and more precise diagnosis arises with the advent of first treatments [3]. Current treatments of AD may in some cases reduce disease progression, but certainly do not reverse the disease process. In addition, cholinesterase inhibitors or glutamate antagonists are currently applied to only a small fraction of patients [35].

The study focused on the analysis of proteins/peptides in CSF obtained from patients presenting to a memory clinic and suffering from various dementias. CE-MS does not require specific antibodies or specific knowledge about the peptides (e.g. solubility, binding or modifications) and yields a comprehensive display of the peptide populations present in CSF without being subject to selection-biases inherent to alternative proteomic methods like matrix-assisted techniques. The high sensitivity (detection limit in the low fmol to amol range) and reproducibility of the CE-MS technology enables the reliable analysis of >800 different peptides in CSF with molecular weights ranging from 0.8 kDa to about 20 kDa.

### Disease specific peptide pattern

From the vast pool of peptides present in CSF, specific biomarkers and subsequent diagnostic models for AD and FTD could be established. The availability of proteome data from patient samples with clear clinical diagnoses enabled the definition of peptides that apparently allow the differentiation of dementias from healthy controls and the differentiation between dementias like AD and FTD in the blinded dataset with high sensitivity and specificity. The MCI data indicate that CE-MS can be utilized to identify incipient AD cases in MCI. Hence this approach may be used for stratification of patients in therapeutic intervention trials. Currently, clinical MCI trials of antidementiva are impaired due to low conversion rates and the inclusion of many patients that will not develop AD in the clinical course. In consequence many MCI trials fail or long term follow up of large patient cohorts are required to reach an acceptable statistical power, making such trials expensive and difficult in design [36].

As expected from earlier attempts to define biomarkers for AD [3], [37], [38], and as evident from the AUC values shown in **Table 2**, single biomarkers are often of limited diagnostic value. However, the classification model based on a combination of distinct and clearly defined single biomarkers shows a high discriminatory value that cannot be reached when using single biomarkers alone. Already our first attempt to use peptide patterns for diagnostic purposes in a prospective natural clinical sample yielded sensitivity and specificity for AD diagnosis in the range that established methods like the measurement of T-tau, P-tau181, and beta-amyloid1–42 can reach only when combined. Fully developed, the new method should show its strength especially in the differential diagnosis of unclear cases. Having the potential to gather the information included in a multitude of peptides instead of detecting a single marker only, valuable and limited CSF samples can be analyzed more efficient and in-depth. This is in line with other studies, where e.g. levels of CSF beta-amyloid1–42 were lower in AD patients and levels of CSF tau were elevated in AD patients [39] and only the combination of these biomarkers allowed the detection and differentiation of AD from healthy controls with a sensitivity and specificity of greater than 80%. To date, the specificity to differentiate AD from other dementias especially in naturalistic samples, like in our prospective sample, is often considerably lower [3], [4]. Similar approaches like the combination of classical AD markers were recently reported with the aim to identify incipient AD cases in patients suffering from MCI [38] or to predict cognitive decline in nondemented older adults [40]. In addition, Hansson et al. described that concentrations of T-tau, P-tau181, and beta-amyloid1–42 in CSF are associated with future development of AD in patients with MCI [41]. The application of CE-MS allowed the definition of a broader array of yet partially unknown biomarkers, which may have the potential of a more refined and earlier detection of the onset of AD. Interestingly our data also indicate an overlap in FTD and AD pathology especially in patients with primary progressive aphasia, which is line with recent observations made in brain biopsies [42]. This finding could have direct therapeutic impact. While there is currently no treatment option for FTD patients, the presence of AD pathology in this FTD subtype could justify the use of the available AD treatments.

A related approach using several unspecific blood plasma markers for inflammation and apoptosis processes in an ELISA array to identify AD claimed worldwide attention [43], but failed to work [44].

### Identification of new AD-biomarkers using CE-MS

To date, we were able to sequence 279 from the 1104 reliable measured peptides and identified 57 peptides of the primary list of 131 potential AD-biomarkers (**Table 4**) present in cerebrospinal fluid of AD patients. The prevalence of sequences among these potential AD markers related to bioactive peptides derived from prohormones normally stored in neuronal dense-core vesicles is striking. In general, AD patients show higher detection frequencies and amplitudes for these synaptic marker peptides in CSF that could point toward alterations in vesicle maturation and transport in AD.

Cognitive decline in dementias is due to loss of synapses or synaptic functionality meaning loss of neurones, loss of synapses on still functioning neurones, or loss of function on still available synapses. Therefore biomarkers of synaptic function and/or damage or complementary changes in the brain, e.g. immunological responses or cellular stress may be especially suited as AD biomarkers with face validity [45]. Our method identified a set of AD biomarkers that are of synaptic origin: Long known suspects like chromogranin A [37] and some newer like neurosecretory protein VGF (VGF), clusterin (apolipoprotein J), ProSAAS, testican-1, and neuroendocrine protein 7B2. We also found fragments of proteins well known to be associated with AD like the amyloid beta peptides, one fragment of beta-amyloid1–42 and one fragment of a soluble form of APP, one fragment of APLP2 and apolipoprotein E [6], [32]. Maybe most interestingly, we found 6 APLP1-derived ‘A-beta-like peptide’ species [46] among them APL1β28, as putative AD biomarkers with our approach. This non-amyloidogenic peptide fragment is proposed to be a CSF surrogate marker of beta-amyloid production [47], [48] and we also identify them as potential AD biomarkers using a different methodology.

So far we were able to identify 5 biomarkers of our diagnostic AD panel of 12 peptides used in this study:

A fragment of neurosecretory protein VGF was identified. For VGF a role in the regulation of energy balance is currently discussed and knockout mice are thin and hypermetabolic [49]. One may speculate whether there is a connection with the heavy weight losses observed in some AD patients. Furthermore, other proteomic approaches have also found decreased VGF as potential biomarker in CSF of AD patients [9].

Phospholemman (FXYD1) is a 72-residue protein, which is expressed in the CNS, e. g. in the choroid plexus and cerebellum. It is a homolog of the Na+/,K+-ATPase y subunit (FXYD2), a small accessory protein that modulates ATPase activity. It forms ion channels selective for K+, Cl−, and taurine in lipid bilayers and colocalizes with the Na+/K+-ATPase and the Na+/Ca2+-exchanger, which may suggest a role in the regulation of cell volume [50], [51]. Na+/K+-ATPase activity might be altered by amyloid in AD [52]. Interestingly, we also found a fragment of FXYD6 (Phosphohippolin).

Clusterin (also called apolipoprotein J) is a secreted glycoprotein. In the central nervous system, clusterin expression is elevated in neuropathological conditions such as AD, where it is found associated with amyloid-beta (Aβ) plaques. Clusterin also coprecipitates with Aβ from CSF, suggesting a physiological interaction with Aβ and can be found in Lewy Bodies [53]. A recent study using an ELISA found that clusterin is significantly increased in cerebrospinal fluid from Alzheimer patients, but concluded that due to individual overlap between the two groups cerebrospinal fluid clusterin measurement is not suitable as a biochemical marker in the diagnosis of AD [54]. With an AUC value of 0.81 derived from the ROC curve our findings suggest that maybe depending from the technique used clusterin fragments might be useful as AD marker. Two recent studies using 2D gel electrophoresis also found increased levels of clusterin in patients with AD [55], [56]. A genome wide association study also identified clusterin as a risk gen for AD [57].

Chromogranin A, a prohormone that is a major ingredient of large dense core vesicles in neurones, is proteolytically processed into low molecular weight peptides in neurons prior to axonal transport and released into the synaptic cleft where they may act as neurotransmitters. Up-regulation of chromogranin A was reported in AD, it is found in beta-amyloid plaques in AD brain biopsies [58], [59] and was proposed to be a marker for synaptic degeneration [37]. Chromogranins are soluble glycophosphoproteins capable of activating microglial cells and metabotropic glutamate receptors [60]. Chromogranin A might synergistically enhance with beta-amyloid peptides the microglial neurotoxic effect and diminish microglial phagocytic activity in senile plaques [61]. Chromogranin A was recently discovered to bind to mutant superoxid dismutase activity in amyotrophic lateral sclerosis increasing neurotoxicity [62]. Furthermore, chromogranin A fragments may be therefore stimulators of senile plaque development and neuronal toxicity and their concentration changes during AD-treatments could be used as surrogate markers. Compared to initial findings [37] chromogranin A fragments maybe contain more information about the disease process than the intact prohormone and may yield better AD biomarkers. Interestingly, beside 7 chromogranin A fragments, we also found fragments of secretogranin I–III.

Proprotein convertase subtilisin/kexin type 1 inhibitor (proSAAS) a recently discovered prohormone is found in synaptic vesicles [63]. N-terminal proSAAS fragments are found in the Tau inclusion bodies of Pick disease and other tauopathies [64], [65]. ProSAAS is distributed abundantly in neuroendocrine tissues like pituitary and hypothalamus and other brain regions and is co-localised with prohormone convertase 1 (PC-1). The C-terminal fragments are potent inhibitors of PC-1 belonging to a family of calcium-dependent serin proteases, the major endoproteolytic processing enzymes of the secretory pathway which are responsible for the proteolytic cleavage of a wide variety of peptide precursors including proinsulin [63], [66]. Endogenous binding and inhibiting proteins for PC-1 and PC-2 have been identified as proSAAS and neuroendocrine peptide 7B2, respectively. A recent study using the iTRAQ technique identified proSAAS and chromogranin A fragments as putative AD markers [67]. ProSAAS was also identified as potential AD marker in the study of Finehout and colleagues [55]. So far we identified 7 fragments of proSAAS to be putative AD biomarkers in our approach.

Most interestingly some of the identified potential AD markers like testican-1 and proSAAS are inhibitors of proteases or like neuroendocrine protein 7B2 are required for the function of prohormone convertases. Recent research has demonstrated the critical importance of such protease inhibitors and their neuroproteases for the regulation of specific peptide neurotransmitters and for the production of toxic peptides in major neurodegenerative diseases like AD [68]. Several of the identified biomarkers are involved in vesicular transport and processing of synaptic neuropeptides. A disturbance in maturation and degradation processes of synaptic neuropeptides may be one reason for the cognitive malfunction and subsequent neuronal loss. One may speculate whether abnormal proteolysis caused by enhanced protease inhibitor activities due to the synaptic peptides identified in this study in AD patients might play a role in cognitive impairment and AD pathology well before tangle and plaque formation occur [69]. Hence, proteases like prohormone convertases could become new drug targets in the development of AD treatments. That we do not only identify new biomarkers but also established markers of AD like fragments of amyloid-beta peptides or currently discussed AD-markers like the APLP1 fragments points to the validity and high potential of our method. A limitation of our study is surely the lack of any neuropathological confirmation of our clinical diagnoses that could help to improve the specificity and sensitivity of our pure clinical approach. When fully developed and validated, this powerful new technique, CE-MS, may be especially suited to monitor drug effects on a synaptic level. To date the method already allows the early diagnosis of AD and differential diagnosis of other dementias showing promising practical advantages in respect to current diagnostic approaches like e. g. ELISAs and PET. New analytical proteomic technologies like ours are now becoming mature and can be applied to clinical problems in dementia research like diagnostic and therapeutic biomarker discovery as more and more studies prove [7], [43], [55], [67].

## Supporting Information

Figure S1 **Reproducibility of the CE-MS measurements.** Protein contour plots of 4 CSF-samples obtained by fractionated extraction of six aliquots of cerebrospinal fluid from one patient. The molecular mass (in kDa, logarithmic scale) on the *y*-axis is plotted against CE-migration time (in min) on the *x*-axis.
(TIFF)

Figure S2 **Comparability of the CE-MS measurements.** (**A**) Contour plot of the entire cerebrospinal fluid proteome. The molecular mass (in kDa, logarithmic scale) on the *y*-axis is plotted against CE-migration time (in min) on the *x*-axis. The arrangement of the peptides in distinct lines is obvious. (**B**) Contour plot of 279 identified peptides. The lines already observed in (**A**) could be comprehended as a result of the number of positive charges z (at pH 2). (**C**) By means of several examples for determined peptide sequences the correlation between the effective netto-charge, molecular mass and the CE-migration time is demonstrated. Basic amino acids are colored in red.
(TIFF)

Figure S3 **Tandem mass spectra of the AD biomarkers.** The mass spectra of all 12 AD biomarkers (see Protein ID) are shown in the following figures. Spectra of the identified peptides are annotated with fragment assignments from the OMSSA (Open Mass Spectrometry Search Algorithm; http://pubchem.ncbi.nlm.nih.gov/omssa) searches. The corresponding sequence is displayed above each spectrum. Identified b-ions are marked in blue and y-ions in red color.
(PDF)

Table S1 **AD marker list.** Initial set of 131 putative AD biomarkers present with high frequency in CSF of AD are listed. Shown are unique internal protein ID, molecular mass (in Dalton), CE-migration time (in minutes), observed frequency of occurrence and the corresponding mean amplitudes in AD and controls, and unadjusted p-values. Peptides that have an unadjusted p-value limit of 0.01 are highlighted in bold letters.
(XLS)

Table S2 **Prospective sample data.** Patients (n = 100): Final diagnoses (clinical outcome) before unblinding: AD (52), still MCI (14), mixed dementia (6), vascular dementia (5), FTD (6), depression (6), schizophrenia (3), delusional disorder (1), encephalitis (2), Parkinson dementia (2), alcohol psychosis (1), amyotropic lateral sclerosis (1), Chorea Huntington (1). Scores: cut-off for AD>0, FTD>−0.1, schizophrenia>0.1. Row AD-MS and AD-IM contains the values for AD diagnosis for mass spectrometry and immunochemistry: tp, true positiv; tn, true negative, fp, false positive, fn, false negative for diagnosis AD. File further includes data for gender, age, diagnoses for the prospective patient sample, scores for peptide patterns and the values for the biomarker total tau, phospho_181_-tau, and beta-amyloid1–42 if available.
(XLS)

Table S3 **Neuropsychological tests used to classify MCI according to Petersen et al.** [**70**] **and dementia cases.** Mini-Mental State Examination (MMSE) [71], Clinical Dementia Rating (CDR) [72], CERAD test battery [73], WMS-R Logical Memory [74], Trail Making Test (TMT) [75], and Clock Drawing Test (CDT) [76].
(XLS)

Table S4 **Precision data examples.** Precision study for ten cerebrospinal fluid samples which are measured twice.
(XLS)

Table S5 **CE-MS raw data of the 176 samples used in the study.** Only data that were actually utilized (that are present in at least 40% of the samples of one of the diagnostic groups, see section “Methods”) are shown. The protein IDs of all peptides are given in the first column named “protein ID”, the unique sample IDs constitute the first row. The MS data from each sample are reflected in one column. The number in each cell represents the normalized signal intensity of the mass spectrometric signal of each peptide detected in the sample.
(XLS)
