# Supplementary figures and images for: Peptide Fingerprinting of Alzheimer's Disease in Cerebrospinal Fluid: Identification and Prospective Evaluation of New Synaptic Biomarkers
Source: PLoS One. 2011 Oct 26;6(10):e26540. doi: 10.1371/journal.pone.0026540 (PMC3202544; doi:10.1371/journal.pone.0026540)

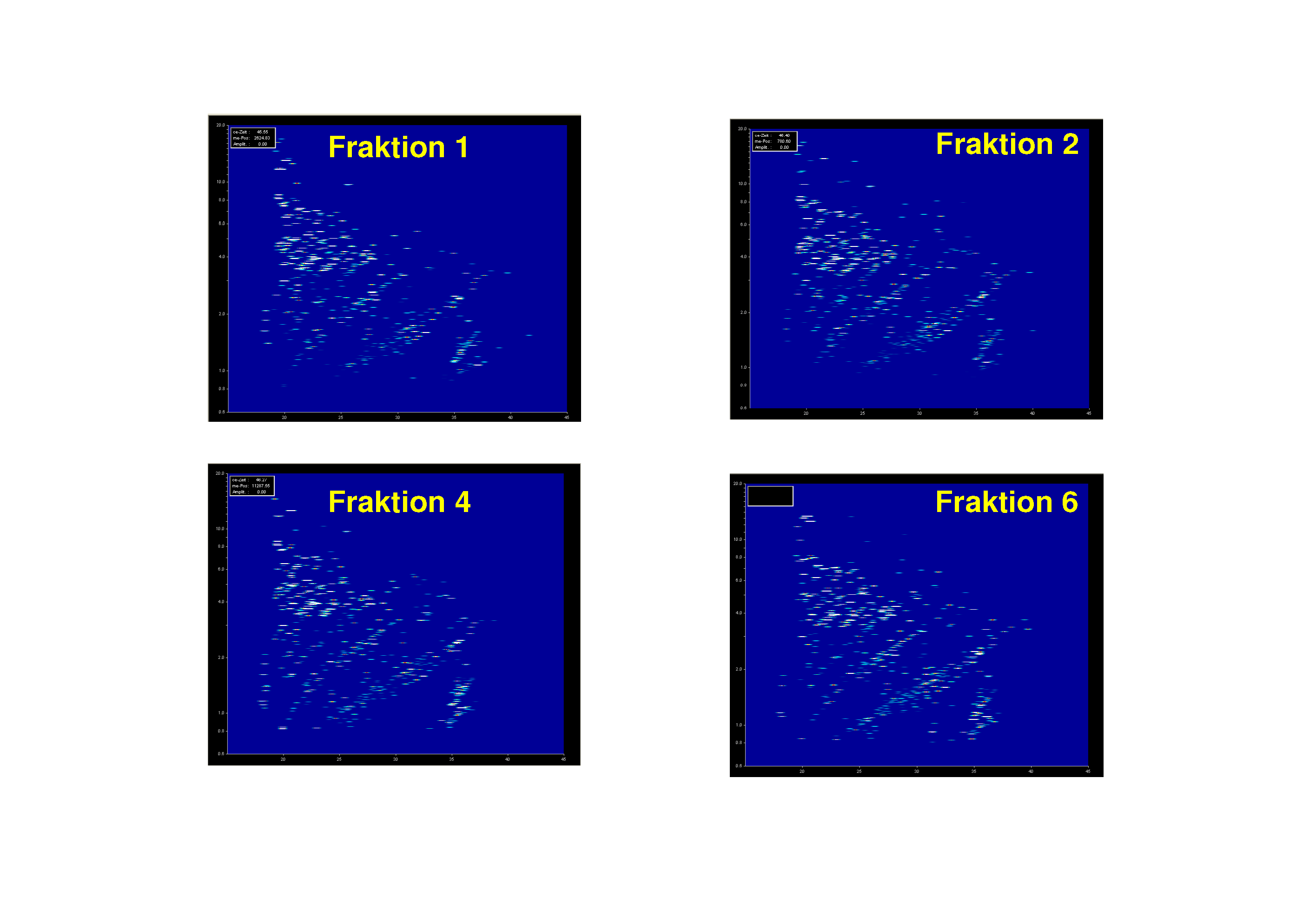

Supplement: Figure S1 — Reproducibility of the CE-MS measurements. Protein contour plots of 4 CSF-samples obtained by fractionated extraction of six aliquots of cerebrospinal fluid from one patient. The molecular mass (in kDa, logarithmic scale) on the y-axis is plotted against CE-migration time (in min) on the x-axis. (TIFF) [file pone.0026540.s001.tiff]

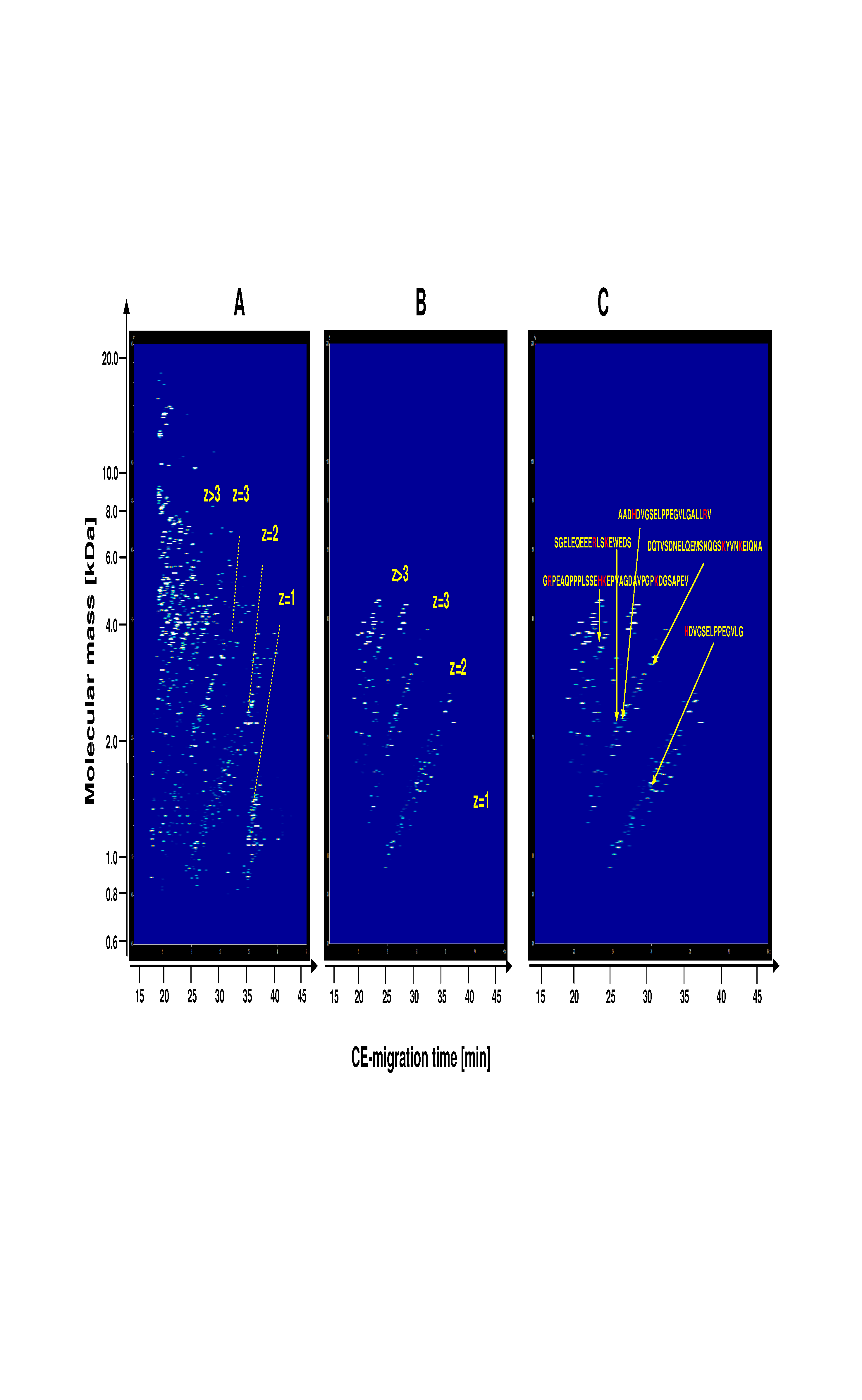

Supplement: Figure S2 — Comparability of the CE-MS measurements. (A) Contour plot of the entire cerebrospinal fluid proteome. The molecular mass (in kDa, logarithmic scale) on the y-axis is plotted against CE-migration time (in min) on the x-axis. The arrangement of the peptides in distinct lines is obvious. (B) Contour plot of 279 identified peptides. The lines already observed in (A) could be comprehended as a result of the number of positive charges z (at pH 2). (C) By means of several examples for determined peptide sequences the correlation between the effective netto-charge, molecular mass and the CE-migration time is demonstrated. Basic amino acids are colored in red. (TIFF) [file pone.0026540.s002.tiff]
