# Supplementary material for: Peptide Fingerprinting of Alzheimer's Disease in Cerebrospinal Fluid: Identification and Prospective Evaluation of New Synaptic Biomarkers
Source: PLoS One. 2011 Oct 26;6(10):e26540. doi: 10.1371/journal.pone.0026540 (PMC3202544; doi:10.1371/journal.pone.0026540)

Protein ID: 11229

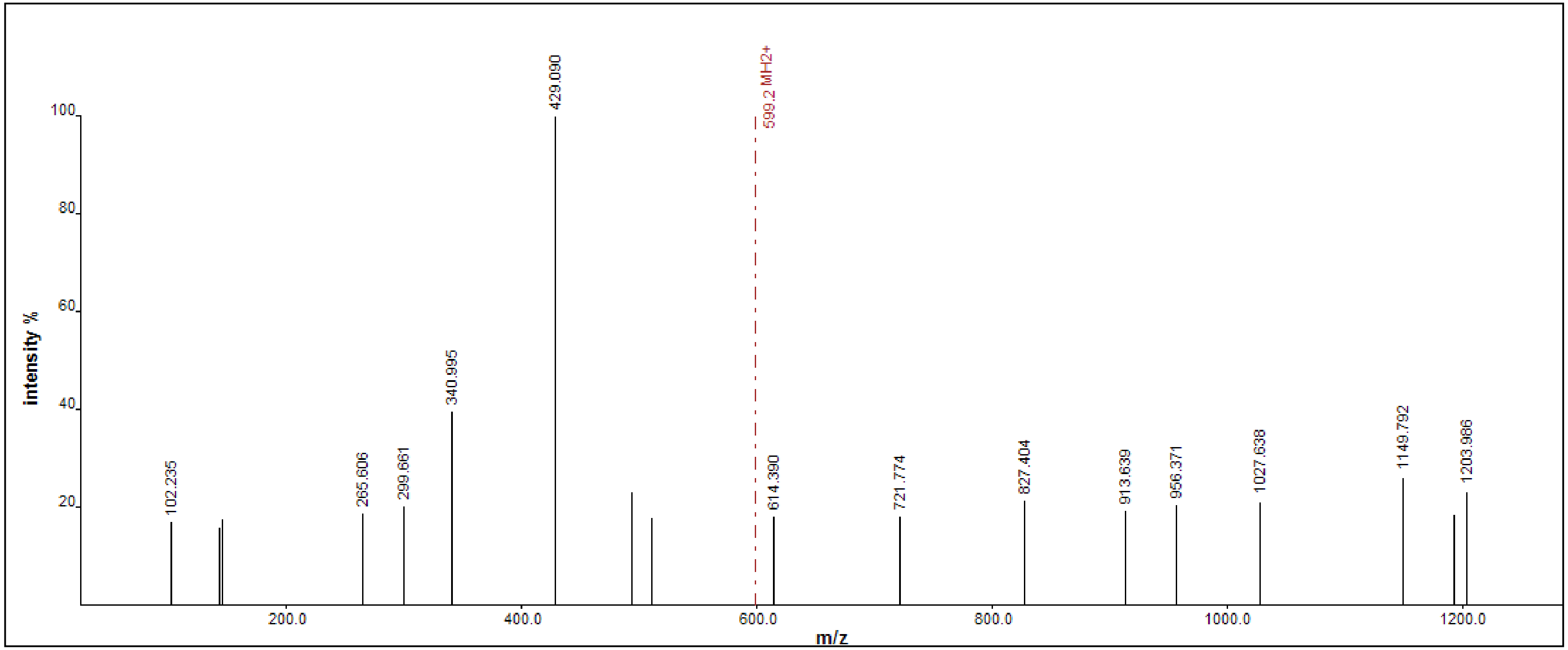

Protein ID: 35146

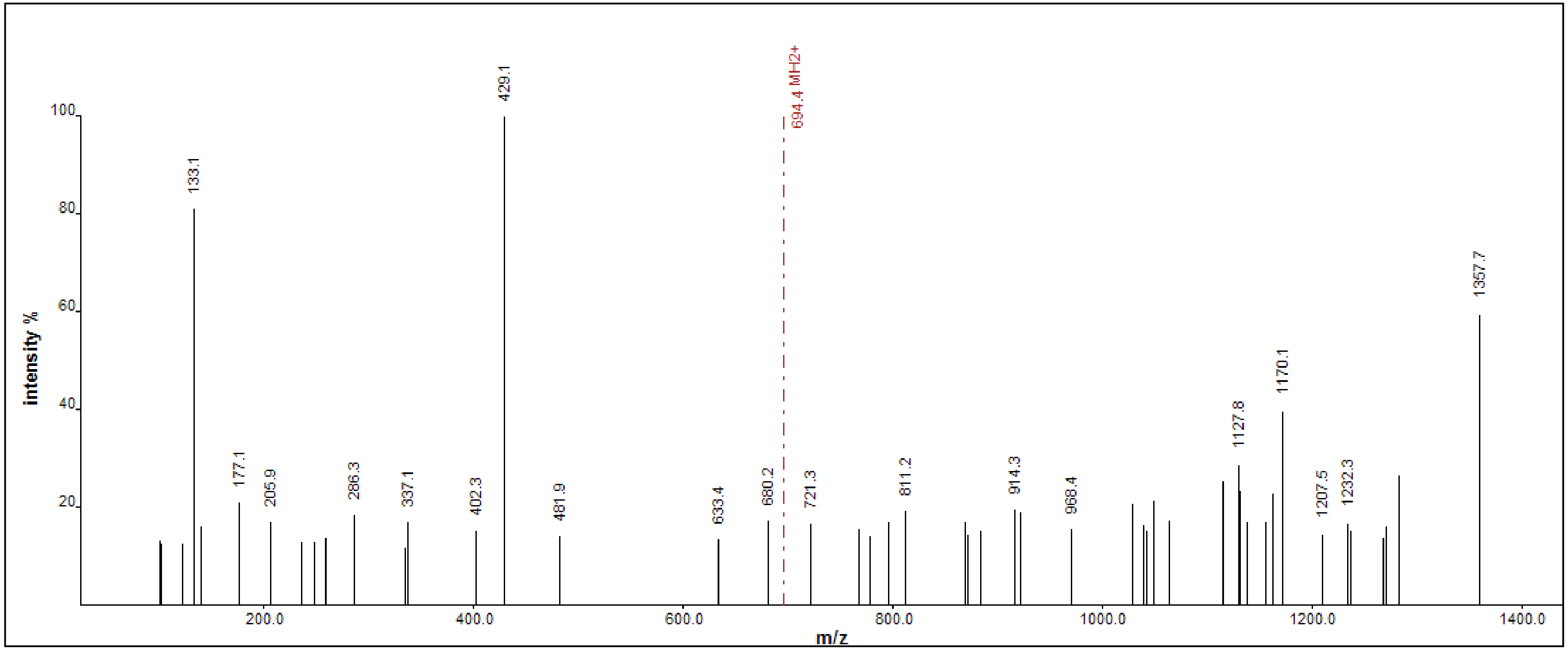

Protein ID: 35998

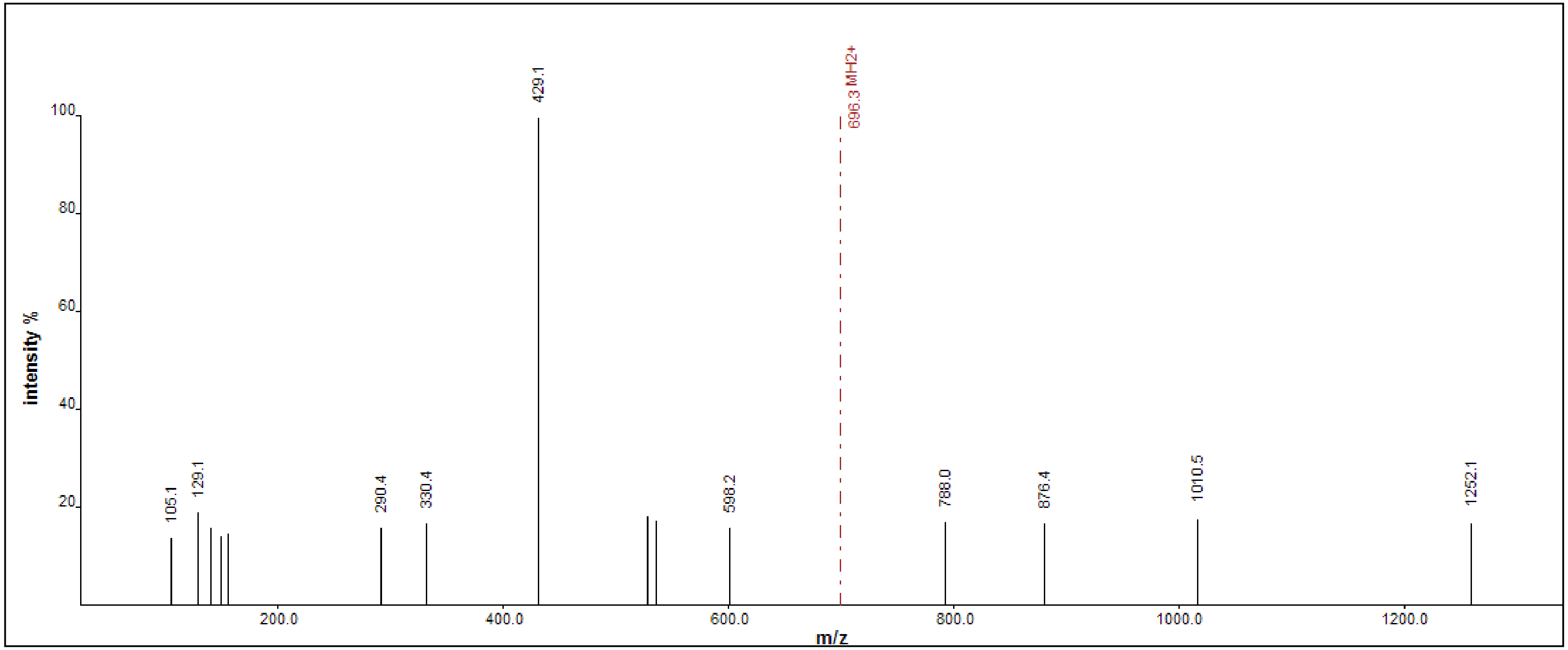

Protein ID: 77519

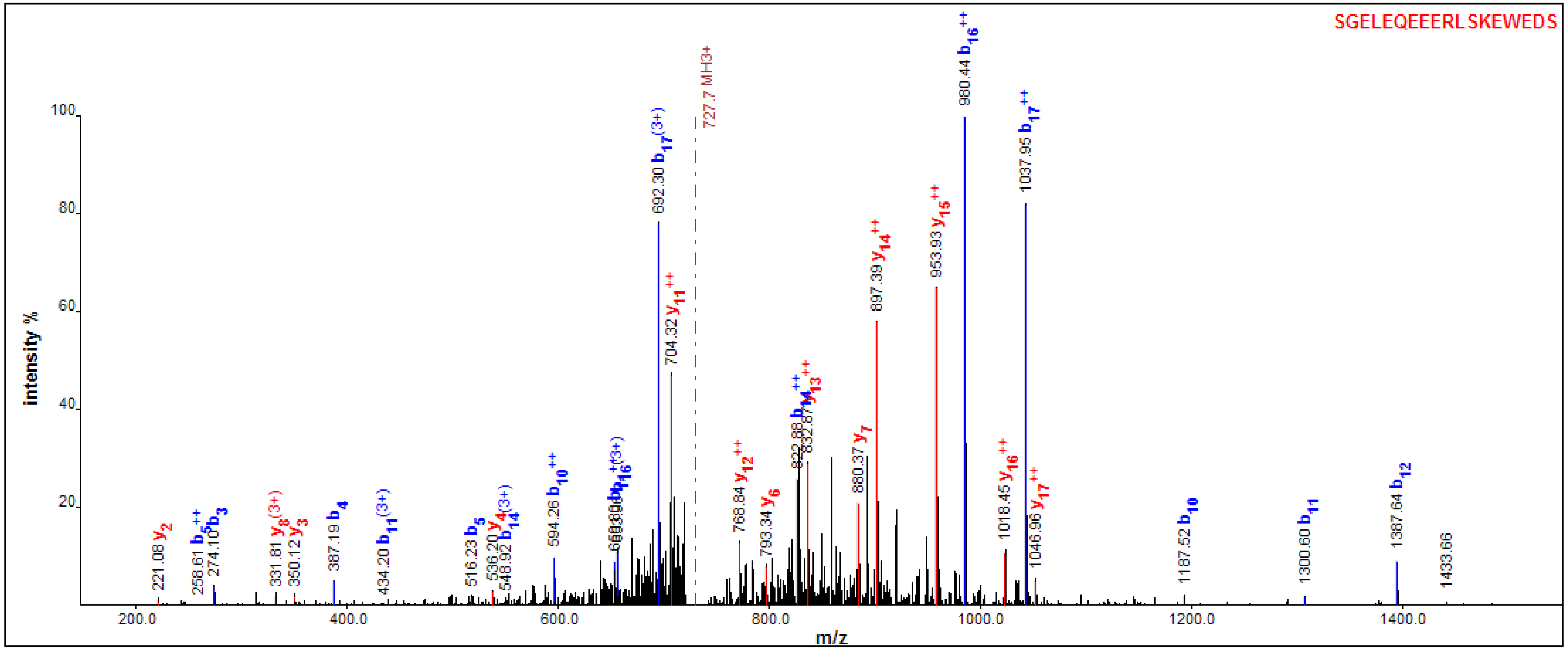

Protein ID: 78842

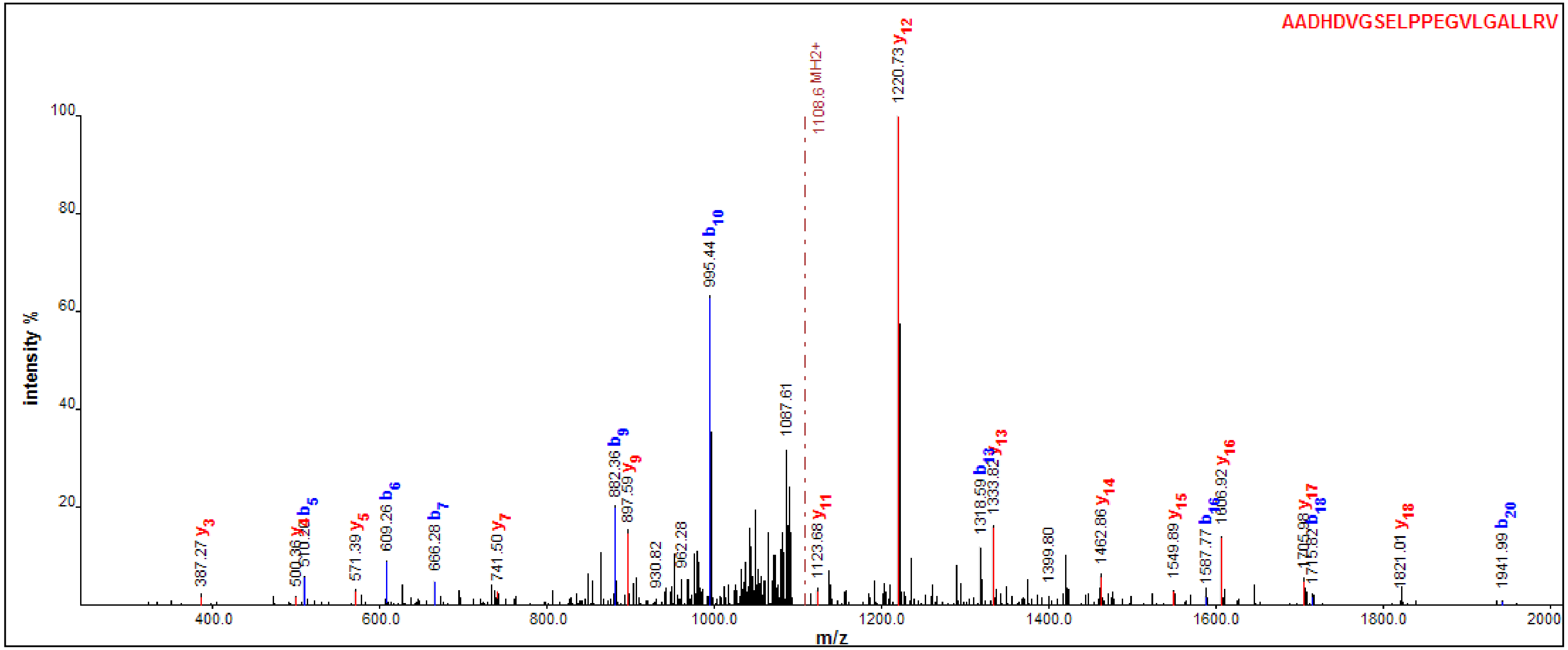

Protein ID: 86638

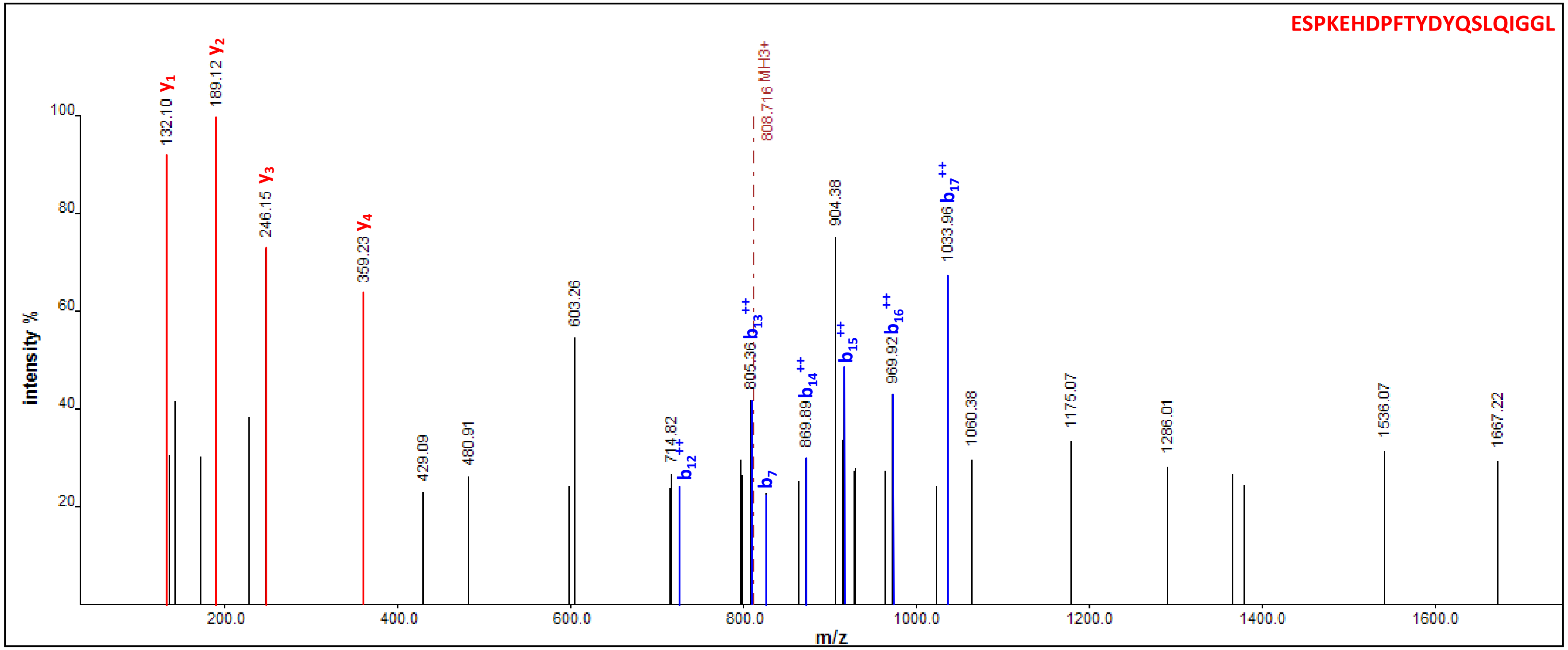

Protein ID: 95324

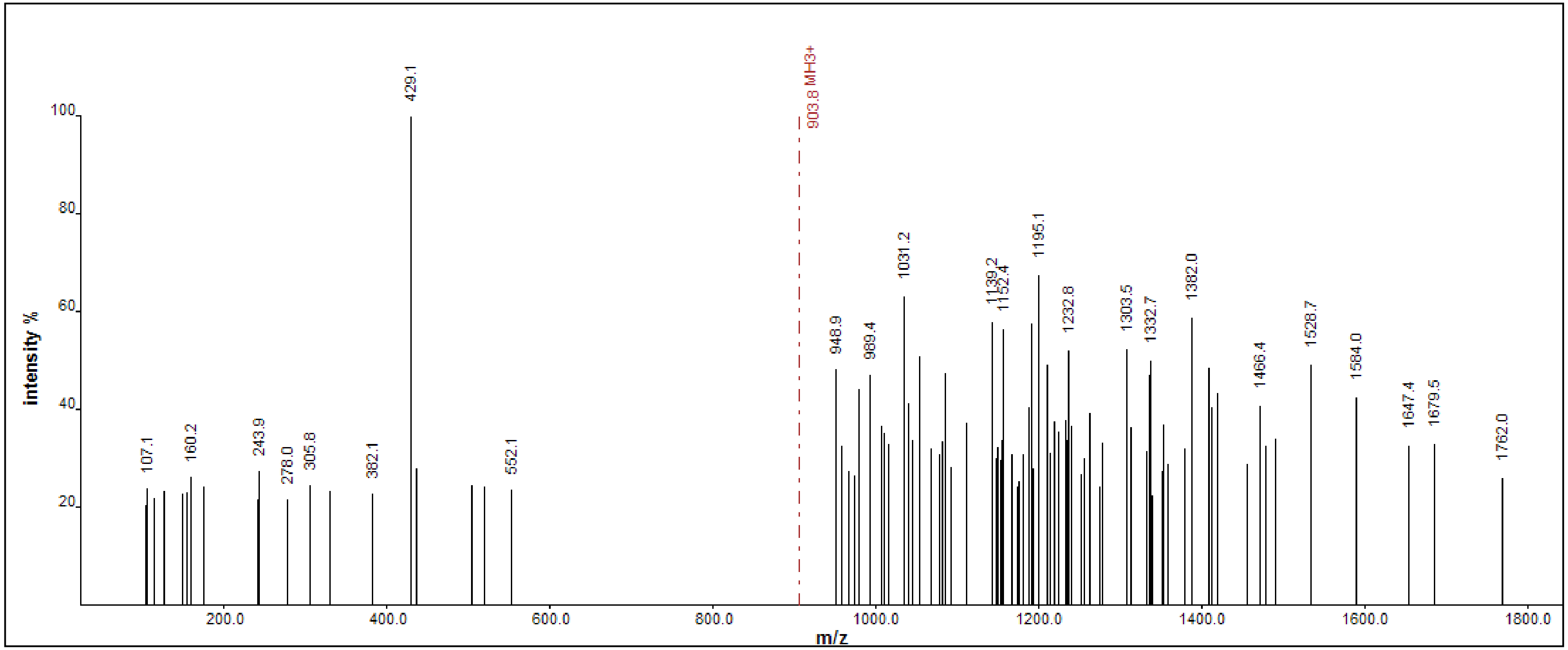

Protein ID: 102634

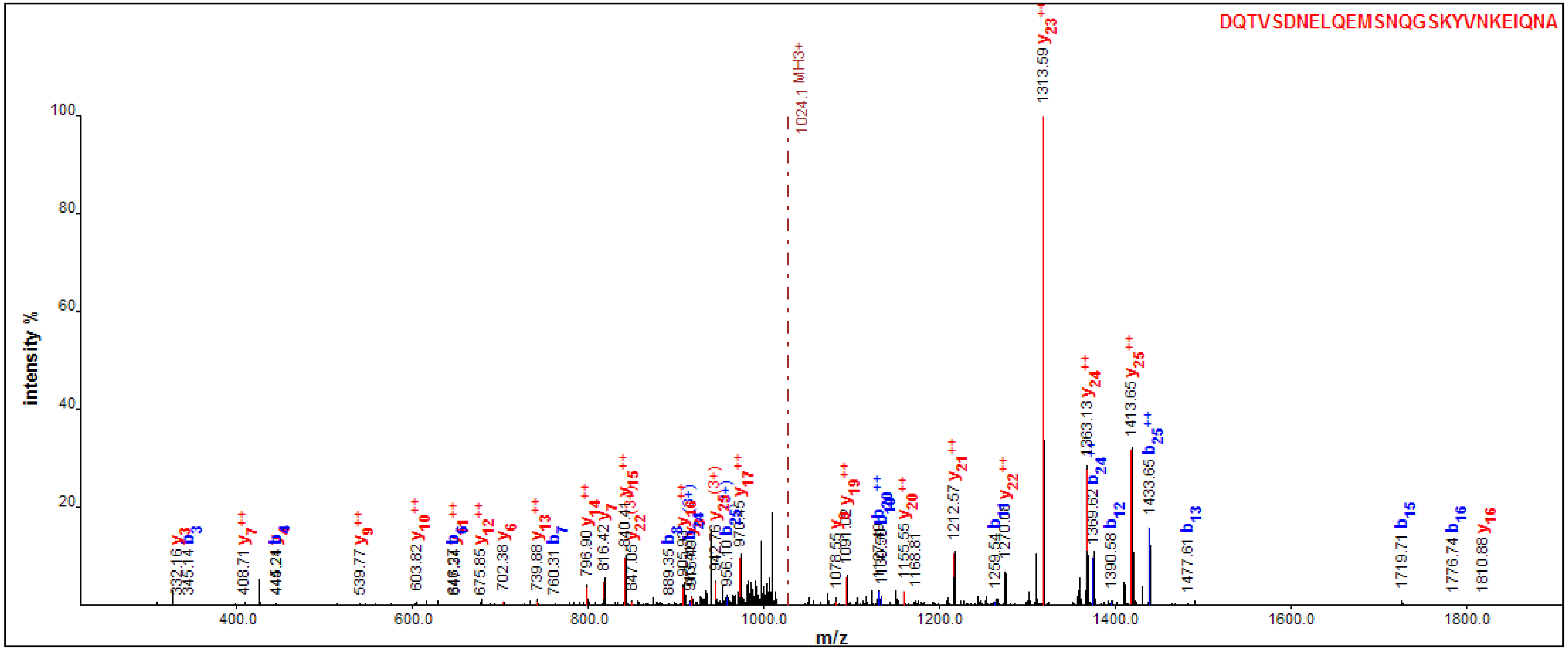

Protein ID: 110596

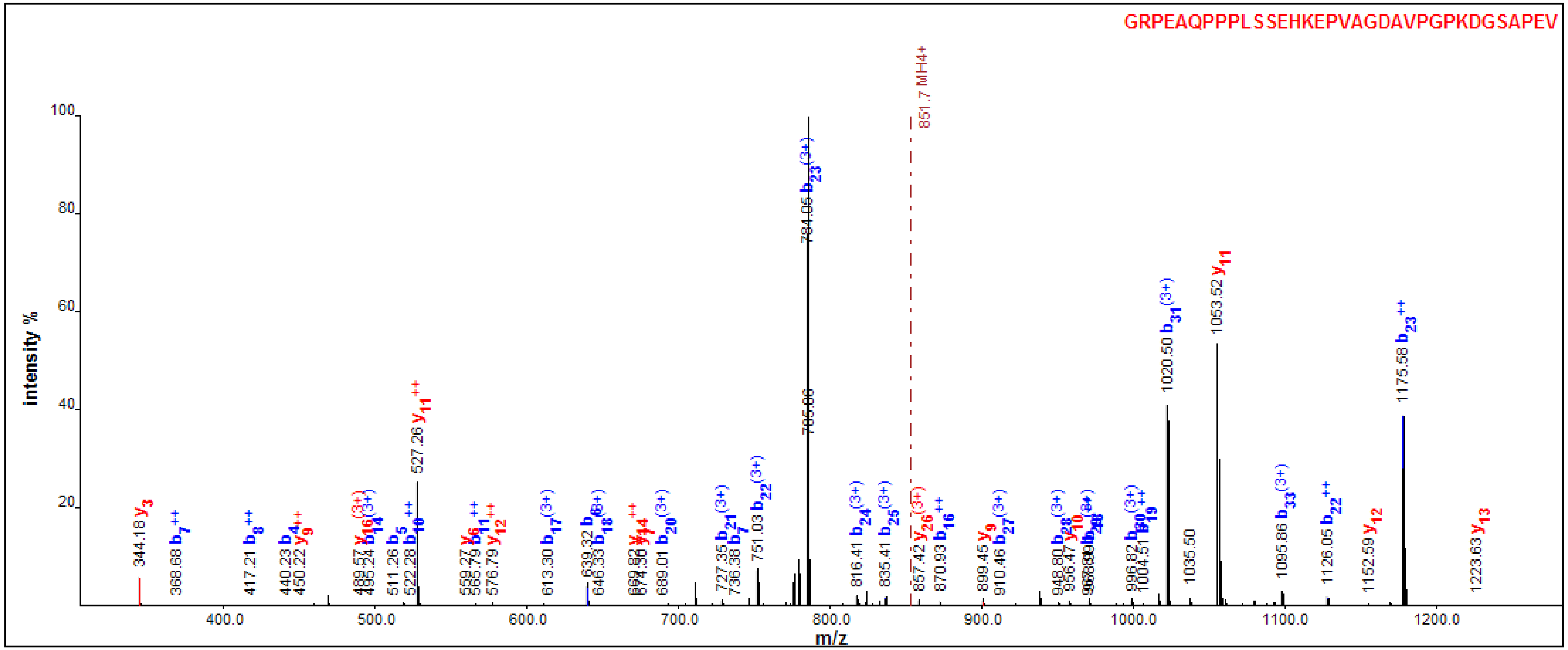

Protein ID: 130889

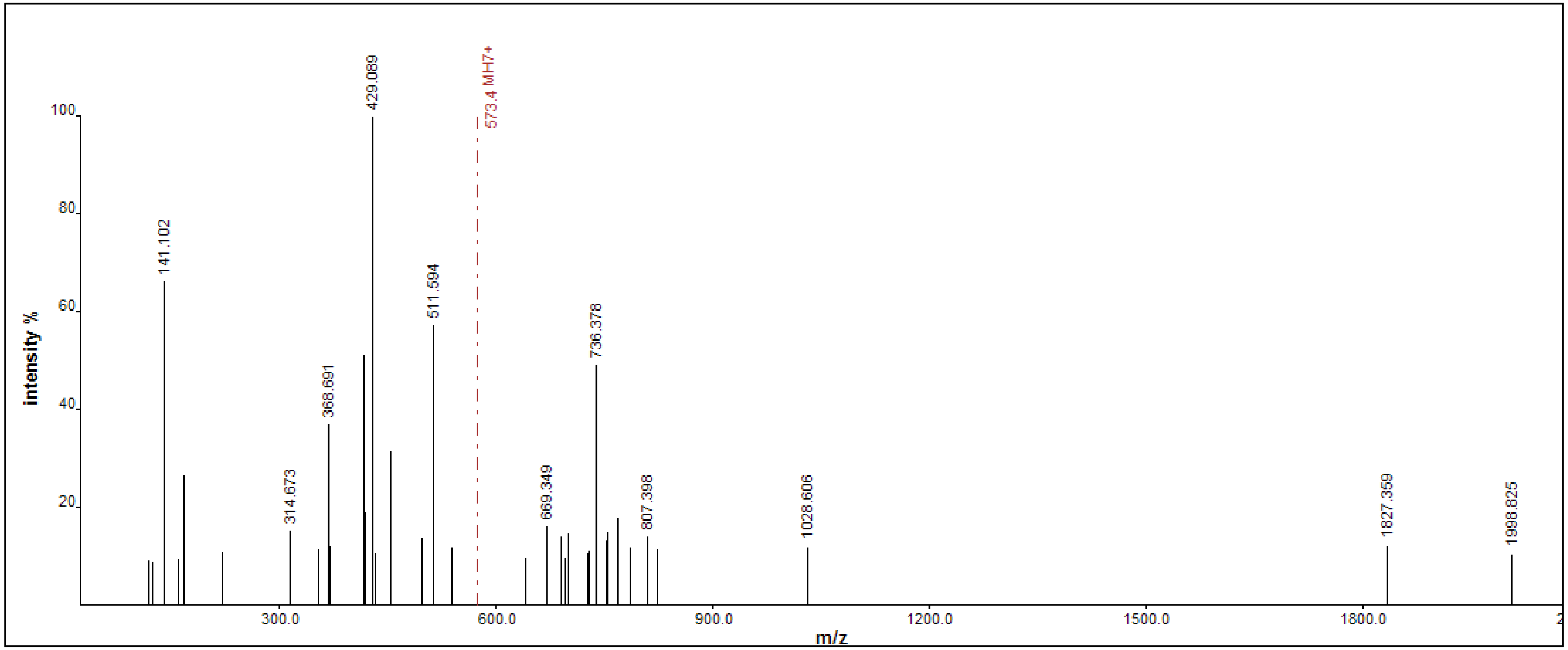

Protein ID: 131863

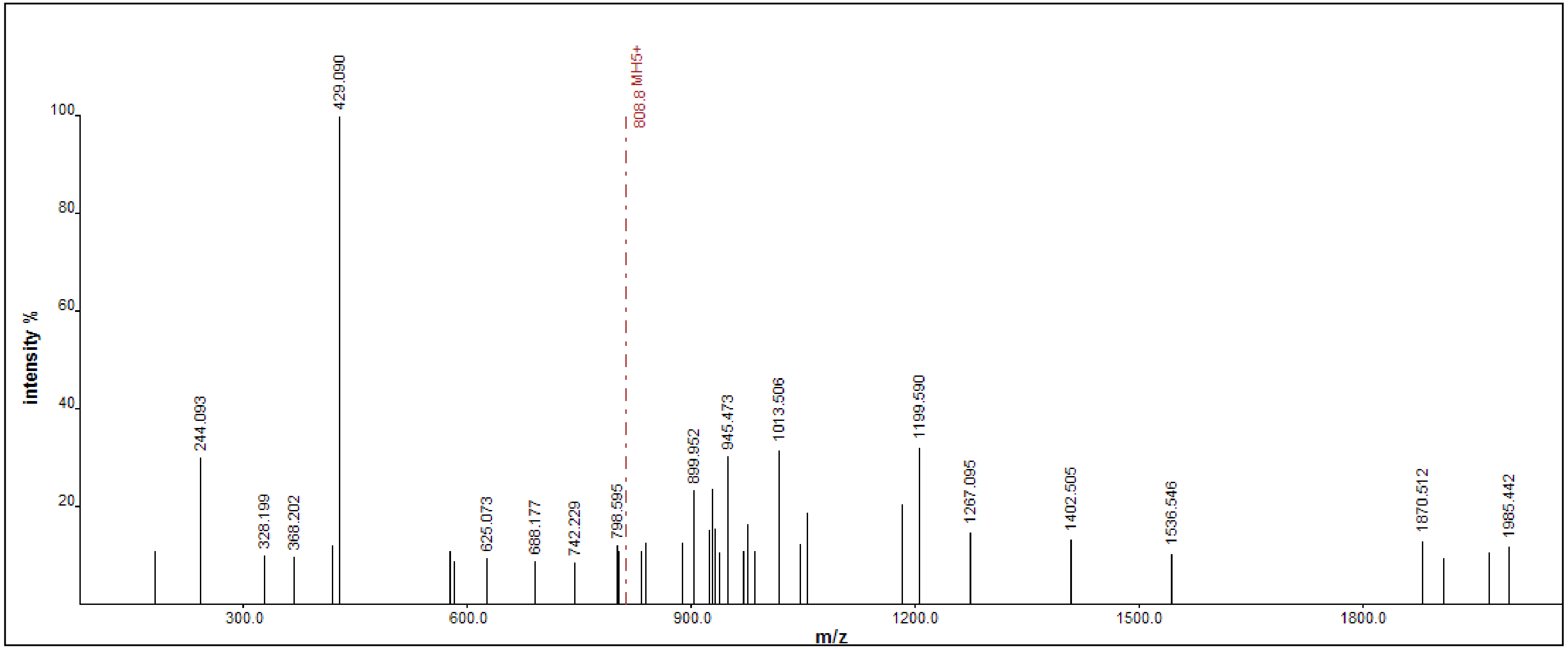

Protein ID: 160599

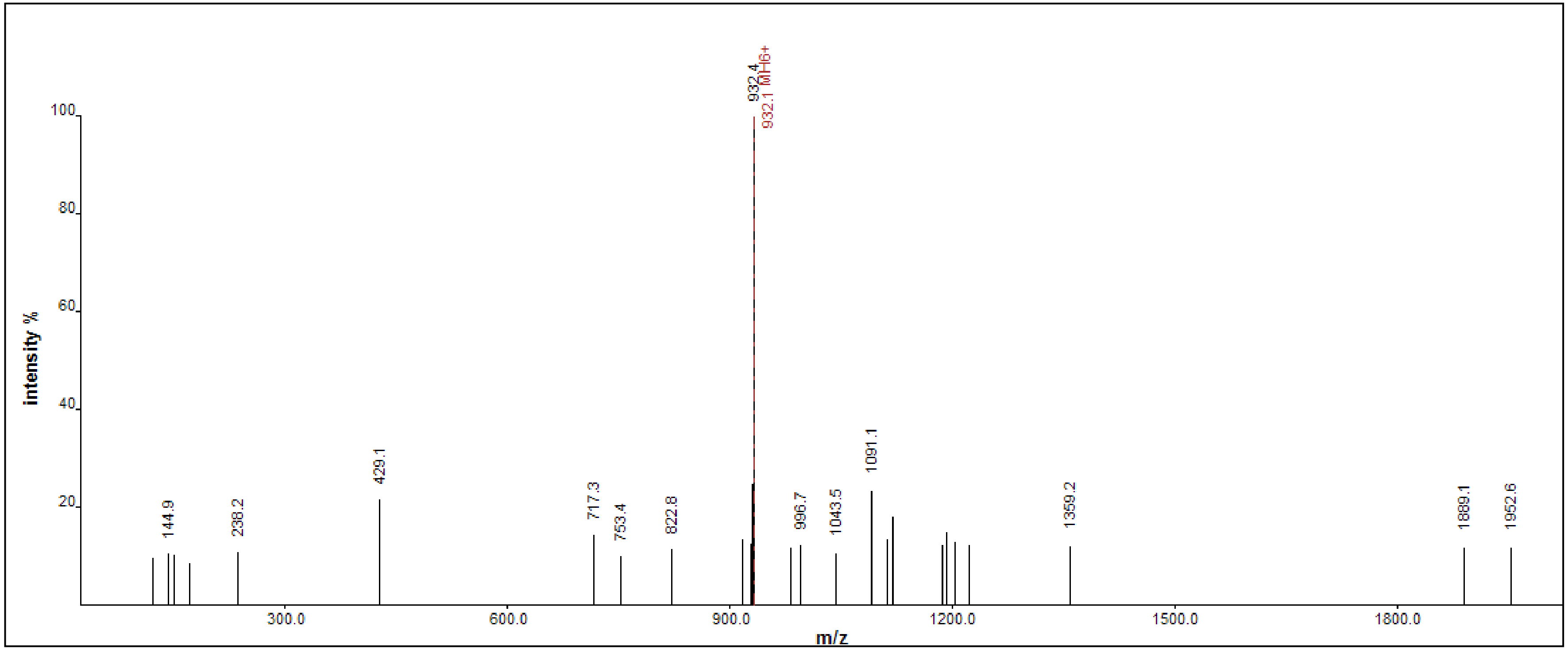

Supplement: Figure S3 — Tandem mass spectra of the AD biomarkers. The mass spectra of all 12 AD biomarkers (see Protein ID) are shown in the following figures. Spectra of the identified peptides are annotated with fragment assignments from the OMSSA (Open Mass Spectrometry Search Algorithm; http://pubchem.ncbi.nlm.nih.gov/omssa) searches. The corresponding sequence is displayed above each spectrum. Identified b-ions are marked in blue and y-ions in red color. (PDF) [file pone.0026540.s003.pdf]
